# Tim‐3 suppresses autoimmune hepatitis via the p38/MKP‐1 pathway in Th17 cells

**DOI:** 10.1002/2211-5463.13148

**Published:** 2021-04-01

**Authors:** Hongwei Wu, Shiyue Tang, Mengya Zhou, Jiji Xue, Zhenjun Yu, Jiansheng Zhu

**Affiliations:** ^1^ Department of Infectious Diseases Affiliated Taizhou Hospital of Wenzhou Medical University Linhai China; ^2^ Department of Pathology Affiliated Taizhou Hospital of Wenzhou Medical University Linhai China

**Keywords:** autoimmune hepatitis, Concanavalin A, MAPK, MKP‐1, Th17 cell, Tim‐3

## Abstract

T‐cell immunoglobulin‐ and mucin‐domain‐containing molecule‐3 (Tim‐3) mediates T‐cell suppression in various autoimmune diseases, such as chronic inflammatory liver disease. However, the regulatory effect of Tim‐3 on Th17 cells in autoimmune hepatitis (AIH) is incompletely understood. Here, we studied the expression and function of Tim‐3 in T cells in AIH patients and in a Con A (concanavalin A)‐induced mouse AIH model. We report that the frequency of CD4^+^Tim‐3^+^ T cells in peripheral blood samples of AIH patients was lower than that in the control group. The p38/MKP‐1 and p‐JNK pathways were activated, and the expression of interleukin‐17A protein was elevated in patients with AIH. Furthermore, the extent of pathological damage in the livers of mice with a blocked Tim‐3 signaling pathway (anti‐Tim‐3 group) was markedly increased and correlated with elevated alanine aminotransferase and aspartate aminotransferase levels. In addition, the frequency of CD4^+^ IL‐17^+^ T (Th17) cells in the anti‐Tim‐3 group was increased, while that in mice with blocked p38 activity was decreased. Finally, the expression of MKP‐1 (p‐p38) gradually increased in the control, Con A, and anti‐Tim‐3 groups, but the levels of interleukin‐17A were decreased in the p38‐blocked group. In summary, our results suggest that Tim‐3 suppresses AIH by regulating Th17 cells through the p38/MKP‐1 pathway.

## Graphical abstract

The regulatory effect of Tim‐3 on Th17 cells in autoimmune hepatitis (AIH) is incompletely understood. In this paper, the expression and function of Tim‐3 in T cells in AIH patients and in a concanavalin A‐induced mouse AIH model were investigated. Our results suggest that Tim‐3 suppresses AIH by regulating Th17 cells through the p38/MKP‐1 pathway.

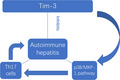

**Abbreviations**
AIH: autoimmune hepatitis
ALP: alkaline phosphatase
ALT: alanine aminotransferase
AST: aspartate aminotransferase
Bat‐3: B‐associated transcript 3
BFA: brefeldin A
CD4 FITC: CD4 fluorescein isothiocyanate
Con A: Concanavalin A
ELISA: enzyme‐linked immunosorbent assay
GGT: gamma‐glutamyltransferase
H&E: hematoxylin and eosin
JNK: JunNH2‐terminal kinase
MCD: methionine and choline‐deficient diet
NTPDase 1: nucleoside triphosphate diphosphate hydrolase 1
PMA: phorbol 12‐myristate 13‐acetate
TBR: total bilirubin
Th1: T helper cell type 1
Tim‐3 APC: Tim‐3 allophycocyanin
Tim‐3: T‐cell immunoglobulin‐ and mucin‐domain‐containing molecule‐3

Autoimmune hepatitis (AIH) is a chronic inflammatory liver disease that can lead to liver cancer and cirrhosis. Many patients with AIH develop acute hepatitis and, eventually, fulminant liver failure [1]. The typical biochemical characteristics of AIH are elevated levels of γ‐glutamyltransferase, aspartate aminotransferase (AST), and alanine aminotransferase (ALT), and normal or slightly higher concentrations of alkaline phosphatase (ALP) [2]. AIH is also signified by the presence of circulating autoantibodies, such as antinuclear antibodies, anti‐mitochondrial antibodies, anti‐centromere antibodies, anti‐smooth muscle antibodies, liver cytosol type 1 antibodies, and type 1 liver/kidney microsomal antibodies [3]. Globally, AIH may occur in children and adults of all ages and races, although most AIH patients are women [4]. There have been reports showing that over the past 24–30 years, the rate of AIH diagnosis has increased [5]. In a recent Swedish cohort study, the prevalence of AIH‐1 was found to be 17.3 cases per 100 000 inhabitants in 2009, compared with 1.2, which had been observed from 1990 to 2009 [6].

CD4^+^ T cells are a group of immune cells required for eliminating pathogens in the body and maintaining long‐term immunity. However, excessive activation of CD4^+^ T cells may be deleterious and lead to immunity‐related diseases, including autoimmune and allergic diseases [7]. During antigen presentation, CD4^+^Th0 cells are activated under the action of appropriate costimulatory signals and differentiate into a variety of T helper cell populations according to the cytokine environment. Th0 lymphocytes differentiate into T helper cell type 1 (Th1) and Th2 under the action of IL‐12 (interleukin‐12) and IL‐4, respectively. Additionally, stimulating factors such as transforming growth factor β and IL‐6 can promote the differentiation of Th0 lymphocytes into Th17 cells to a certain extent [4]. Recent studies have found that immune‐mediated liver injury in AIH is induced by autoreactive CD4^+^ T cells, particularly Th17 cells [8]. Grant *et al*. reported that Treg cells are decreased in frequency and fail to inhibit IL‐17 production by CD4+ T cells in AIH patients [9].

T‐cell immunoglobulin‐ and mucin‐domain‐containing molecule‐3 (Tim‐3) is a membrane protein (type I) comprised of 281 amino acids, with immunoglobulin V‐like and mucin‐like extracellular domains [10]. After the interaction between Tim 3 and its ligand galectin‐9 is established, Tyr 256 and Tyr 263 become phosphorylated and release human leukocyte antigen B‐associated transcript 3 (Bat‐3) from the cytoplasmatic tail of Tim‐3, allowing the latter to bind to the SH2 domain of Src kinases (Lck, Fyn, and others) and subsequently mediate T‐cell suppression [11]. Tim‐3 is involved in several human autoimmune diseases. In mice, anti‐Tim‐3 antibody can aggravate autoimmune encephalomyelitis, which is mediated by T cells [12], and Tim‐3 alleviated methionine‐ and MCD (choline‐deficient diet)‐induced steatohepatitis by reducing production of reactive oxygen species and related pro‐inflammatory cytokines in macrophages [13]. Wang *et al*. reported that the Tim‐3/galectin‐9 pathway regulated the function of CD4+ CD25+ Tregs [14], and it was further found that the defective galectin‐9/Tim‐3 pathway was linked with impaired immune regulation of AIH [15]. In addition, Gal‐9 secreted by hepatocytes could target Tim‐3 and efficiently suppress the intrahepatic T‐cell activation, thus attenuating Th1 response in AIH [16]. These studies suggested the involvement of galectin‐9/Tim‐3/Tregs axis in AIH. In this study, we investigated the regulatory effect of Tim‐3 on Th17 cells and the involvement of p38/MKP‐1 pathway in T cells derived from patients with AIH and also in a concanavalin A (Con A)‐induced murine AIH model.

## Materials and methods

### Reagents

Con A, SB203580, ionomycin, brefeldin A (BFA), and phorbol 12‐myristate 13‐acetate (PMA) were provided by Sigma‐Aldrich (Saint Louis, MO, USA). Human ELISA kits (MKP‐1, IL‐17A, p‐p38, and p‐JNK) were purchased from R&D Systems, Inc. (Minneapolis, MN, USA). Anti‐phospho‐JNK (p‐JNK), anti‐phospho‐p38MAPK (p‐p38MAPK), and GAPDH antibodies were purchased from Cell Signaling Technology (Danvers, MA, USA). Anti‐MKP‐1 antibody was purchased from Sangon Biotech Co., Ltd (Shanghai, China). Anti‐Tim‐3 and anti‐IL‐17A antibodies were purchased from Abcam (Cambridge, MA, USA). Tim‐3 allophycocyanin (Tim‐3 APC), IL‐17A phycoerythrin, and CD4 fluorescein isothiocyanate (CD4 FITC) mouse antibodies and Tim‐3 BB515 and CD4 APC human antibodies were purchased from BD Biosciences (San Jose, CA, USA).

### Animal model and experimental groups

Pathogen‐free male C57BL/6 mice (7 weeks old) with weight of 24–30 g were purchased from Shanghai Jie si Jie Experimental Animal Co. Ltd. (Shanghai, China; license number: SCXK 2018‐0004). Mice were raised in the Public Research Platform Animal Experiment Center of Taizhou Hospital, affiliated to Wenzhou Medical University, at 20–25 °C and relative 60% humidity, with *ad libitum* access to food and water. The experiment started after 1 w of acclimatization. The animal study was approved by the Animal Care and Use Committee of Taizhou Hospital. The study methodologies conformed to the Declaration of Helsinki.

Using a randomized block design, 44 mice were divided into five groups: the control (*n* = 4), Con A‐alone (*n* = 10), Con A+ anti‐Tim‐3 (*n* = 10), Con A+ anti‐IL‐17A (*n* = 10), and Con A+ p38‐blocked (*n* = 10) groups. The mice in the Con A+ anti‐Tim‐3 group were injected with 100 µg of Tim‐3 monoclonal antibody inhibitor (BioLegend, San Diego, CA, USA) intraperitoneally (dissolved in PBS solution), every other day beginning 1 week before the injection of Con A for a total of four injections. Meanwhile, the Con A+IL‐17A received 200 µg of anti‐IL‐17A monoclonal antibody inhibitor (dissolved in dd water) intraperitoneally (BioLegend) 1 day before the injection of Con A. The Con A+p38‐blocked group was injected intraperitoneally with 15 mg·kg^−1^ of SB203580, a p38 inhibitor (dissolved in solution consisting of 4% DMSO, 30% PEG300, 5% Tween‐80, and ddH_2_O) on the day of injecting Con A. For each injection of antibodies or inhibitors, the corresponding vehicles with equal amount were given to mice in other groups. One hour later, all mice except those in the control group were injected with 20 mg·kg^−1^ of Con A (dissolved in saline solution) via tail vein to induce AIH according to the previous literature [17]. The blood, liver, and spleen tissues were collected 8 h later.

### Human sample collection

Thirty‐four human samples were collected from Taizhou Hospital in Zhejiang Province from June to October 2019, including 16 samples from patients with AIH and 18 samples from healthy people as the control group (Table 1). Inclusion criteria were as follows: (a) the score developed by International Autoimmune Hepatitis Group (IAIHG) > 10 [18]; (b) aged 18–75 years; and (c) informed and agreed to participate in this study. Exclusion criteria were as follows: (a) other types of liver injury, Wilson's disease or antitrypsin deficiency; (b) overlap syndrome, including PBC/AIH overlap syndrome and PSC/AIH overlap syndrome; (c) compensated or decompensated cirrhosis; (d) diabetes, gout and phenylketonuria; (e) previous or current tumor; and (f) women who are pregnant or lactating. The protocol of this study was approved by the Institutional Review Board of Taizhou Hospital, and informed consent was signed by all participants.

**Table 1:** Clinical data of normal and AIH groups.

	Control (*n* = 18)	AIH (*n* = 16)	*P*‐value
Age, years	45.2 ± 15.4	58.6 ± 11.5	< 0.01
Male, *n* (%)	6 (33.0)	2 (12.5)	0.233
ALT (U·L^−1^)	14.5 ± 4.5	81.0 ± 60.0	< 0.001
AST (U·L^−1^)	19.1 ± 3.9	56.0 ± 36.5	< 0.001
ALP (U·L^−1^)	72.3 ± 15.0	104.6 ± 34.7	< 0.01
GGT (U·L^−1^)	17.1 ± 8.9	47.8 ± 33.4	< 0.01
TBR (μm)	16.0 ± 3.3	15.6 ± 5.4	0.8381

### Biochemical analysis of serum

Retro‐orbital blood sampling was performed 8 h after injection of the Con A through the tail vein, and the blood was centrifuged at 3000 ***g*** for 10 min at 4 °C. After centrifugation, serum samples were taken for detection of ALT and AST. The levels of ALT and AST were measured using an automatic biochemical analyzer (MODULE P800; Roche, Basel, Switzerland). The serum levels of ALT, AST, ALP, glutamyl transpeptidase (GGT), and total bilirubin (TBR) in patients with AIH and healthy people were also determined by using an automatic biochemical analyzer (MODULE P800; Roche, Basel, Switzerland).

### Histopathological analysis

The samples of liver tissues harvested 8 h after Con A administration were stored in 4% paraformaldehyde for at least 24 h and then embedded in paraffin. Sectioned tissues (4 μm in thickness) were stained with hematoxylin and eosin (H&E). The degree of inflammation and tissue damage was noted under a light microscope. High‐power fields (×200 and ×400) were randomly selected for each slice to observe liver tissue changes. The grading of inflammation was performed based on Scheuer's modified scale (G0‐G4) by two independent experienced pathologists [19]. In addition, the average lymphocyte number in fields under ×400 magnification was used to quantify the staining results.

### Flow cytometry

Flow cytometry was performed to detect the quantitative proportion of CD4^+^ Tim‐3^+^ T cells and CD4^+^ IL‐17^+^ T cells. Murine spleens were collected, and lymphocytes were resuspended with 100 μL of RPMI 1640 medium and incubated with 2.5 μg·mL^−1^ PMA, 1.0 μg·mL^−1^ Ionomycin, and 10 μg·mL^−1^ BFA for 4 h in a humidified incubator with 37 °C and 5% carbon dioxide. In addition, 200 μL aliquots of human peripheral blood samples from each group were diluted with 800 μL of RPMI 1640 medium and incubated with 2 μL Leukocyte Activation Cocktail (BD Bioscience) in a humidified incubator with 37 °C and 5% carbon dioxide for 4 h. After incubating the murine samples with Tim‐3 APC and CD4 FITC mouse antibodies, and human samples with Tim‐3 BB515 and CD4 APC human antibodies for 20 min in the dark at room temperature, the cells were washed twice with PBS buffer, and incubated with 250 μL fixed membrane breaker (BD Bioscience) along with mice samples for 20 min. They were then washed twice with washing buffer (BD Bioscience), incubated with IL‐17 PE mouse antibody for 20 min, and washed twice with PBS buffer. Subsequently, the cells were analyzed using a FACSort cell analyzer (FACSCalibur; Becton‐Dickinson, San Jose, CA, USA) according to the manufacturer's protocol.

### Western blot analysis

Total proteins from mouse livers were extracted by RIPA buffer (provided by Beyotime, Shanghai, China) under the conditions of ice, and concentrations of total proteins were measured by a BCA Protein Assay Kit (provided by Beyotime). The total proteins were purified with high‐temperature pyrolysis before loading, and western blotting was performed as described previously [20]. Proteins (10 μg) were separated by polyacrylamide gel electrophoresis (Sigma‐Aldrich) and transferred to PVDF membranes (Sigma‐Aldrich). The membranes were first incubated with skimmed milk powder (5%) for 2 h at room temperature, and then incubated with antibodies against Tim‐3, IL‐17A, GAPDH, MKP‐1, p38, and p‐JNK overnight at 4 °C. The blots were then incubated for 1 h with horseradish peroxidase combined with goat anti‐rabbit immunoglobulin G (dilution 1 : 5000; cat. no A0208; Beyotime), and protein bands were detected using an enhanced chemiluminescence detection system (provided by Cell Signaling Technology).

### ELISA (enzyme‐linked immunosorbent assay)

The concentrations of inflammatory cytokines and signaling proteins in the supernatants were determined using commercial human ELISA kits: IL‐17A, MKP‐1, p‐p38, and p‐JNK, in accordance with the manufacturer's protocol. Standard curves were drawn using the concentration of standard substance as *x*‐axis and OD value as the *y*‐axis, and the concentration of target protein was calculated by multiplying the concentration calculated from the standard curve with dilution ratio.

### Statistical analysis

spss 22.0 (SPSS, Chicago, IL, USA) was used for all statistical analyses. All data are shown as mean ± SD. We used *t*‐test and one‐way ANOVA to compare parametric variables, which obeyed normal distribution between two and multiple groups, respectively, and the LSD test for pairwise comparison of the means. A *P*‐value < 0.05 was considered statistically significant.

## Results

### Reduced Tim‐3 levels and increased IL‐17A expression in the peripheral blood of AIH patients

**Fig. 1:**
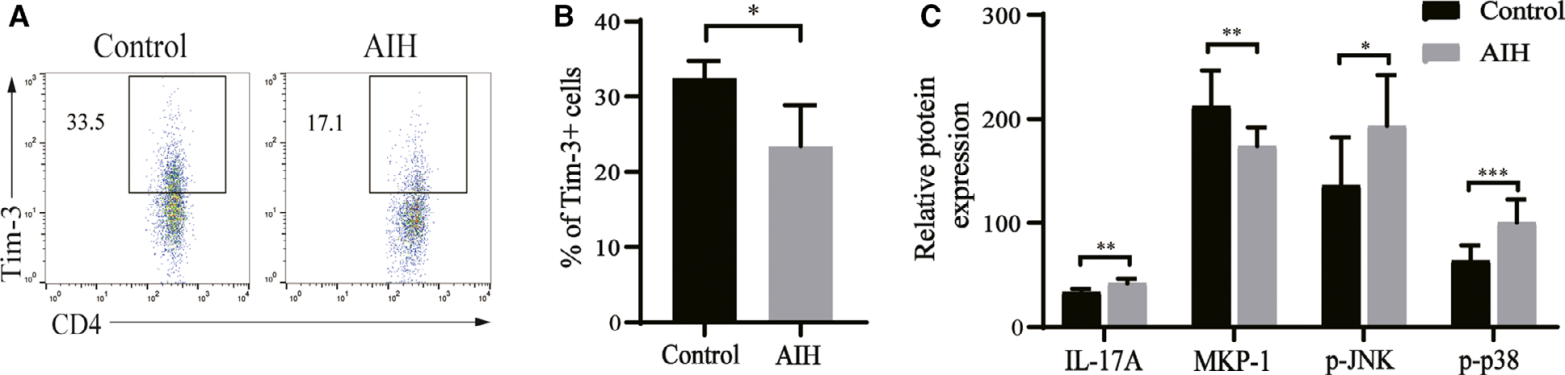
p38/MKP‐1 and p‐JNK pathways were activated, and IL‐17A levels were elevated in AIH patients (*n* = 16) compared with the control group (*n* = 18). (A) and (B) are the frequencies of CD4^+^ Tim‐3^+^ T cells in human peripheral blood. (C) Expression of IL‐17A, MKP‐1, p‐JNK, and p‐p38 in human serum samples. *T*‐test was used for comparison between the two groups. All values are reported as mean ± SD, *P < 0.05, **P < 0.01, and ***P < 0.001. The experiments were performed in triplicate.

The differential expressions of Tim‐3 in CD4+ T cells and related proteins including IL‐17A, p‐p38, and p‐JNK between patients with AIH and healthy people were evaluated and compared. The ALT, AST, ALP, and GGT levels were significantly higher in patients with AIH than in the healthy controls (*P* < 0.01), but there was no statistical difference in TBR levels between the two groups (Table 1). The expression frequency of Tim‐3 on the surface of CD4+ T cells in human peripheral blood tested by flow cytometry was lower in the AIH group (23.4 ± 4.5) than in the control group (32.4 ± 2.0; Fig. 1A,B), indicating that the reduction in Tim‐3 expression is associated with AIH. Additionally, increased IL‐17A, p‐p38, and p‐JNK levels but decreased MKP‐1 levels were detected by ELISA in human serum samples from patients with AIH, as compared to the control group (*P* < 0.05; Fig. 1C). In the AIH group, the serum level of IL‐17 was positively correlated with serum levels of p‐JNK and p‐p38 (*R* = 0.569, *P* < 0.05 for p‐JNK and *R* = 0.535, *P* < 0.05 for p‐p38) and negatively correlated with MKP‐1 (*R* = −0.736, *P* < 0.01).

### Con A‐induced liver damage was aggravated by blocking Tim‐3 and alleviated by blocking p38

**Fig. 2:**
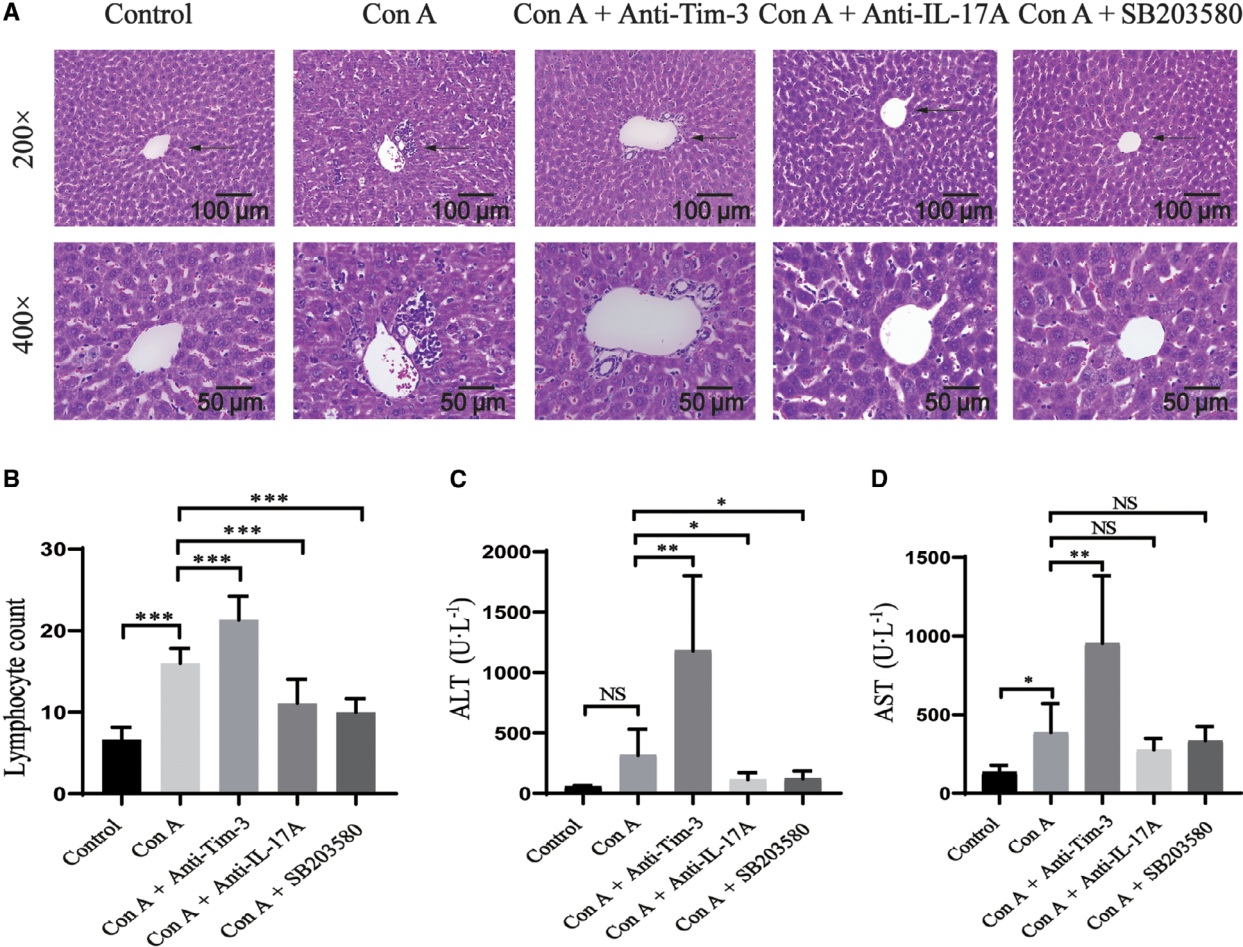
Blocking Tim‐3 function promoted AIH, but blocking the p38 pathway reduced inflammation. After 8 h of Con A induction, mouse liver tissues and serum samples were collected. (A) Hematoxylin‐eosin staining of liver sections, ×200 (scale bar: 100 μm) and ×400 (scale bar: 50 μm); (B) the lymphocyte count of liver sections; (C) (D) ALT and AST levels in mouse serum. One‐way analysis of variance was used to analyze the difference in results between multiple groups, and the LSD test was used for pairwise comparison. All values are reported as mean ± SD, *P < 0.05, **P < 0.01, and ***P < 0.001, and NS indicates no significant difference. The experiments were performed in triplicate.

A murine AIH model was established by the injection of Con A, and H&E staining of liver tissues and the measurement of ALT and AST were performed to assess the effect of blocking Tim‐3 and p38 on liver pathology. The presence of vacuoles and infiltrated lymphocytes and significant damage to liver structure were observed in Con A‐induced AIH mice (Fig. 2A,B). The degree of inflammation and pathological changes (grade G1 to G2), as well as the levels of ALT (315.7 ± 198.9) and AST (385.9 ± 174.0), was significantly higher in the mouse model of AIH than in the controls (*P* < 0.05), indicating that the AIH mouse model was successfully established. Liver samples from the Tim‐3 blocked group, in which anti‐Tim‐3 antibody was used to block the Tim‐3 signaling pathway for 1 week prior to administering Con A, were also analyzed. H&E staining showed obvious lymphocyte infiltration and formation of a large number of vacuoles. ALT (1180.5 ± 567.5) and AST (954.8 ± 392.0) levels were significantly higher in the Tim‐3 blocked group than in the Con A‐alone group (*P* < 0.05). In the p38‐blocked group, inflammatory cell infiltration was reduced, the hepatic lobules were intact, and the liver cords were arranged in a radial pattern. Compared with the Con A group, the levels of ALT (151.6 ± 131.5) were decreased (*P* < 0.05), but those of AST (308.0 ± 116.1) were not (*P* > 0.05) (Fig. 2C,D). The anti‐IL‐17A group showed less inflammatory cell infiltration and lower levels of ALT (123.1 ± 58.5) in liver tissues as compared to Con A alone (*P* < 0.05), but no significant difference in the levels of AST (454.5 ± 325.8) (*P* > 0.05).

### Higher levels of Th17 cells and lower levels of CD4^+^ Tim‐3^+^ T cells and Th17 cells after blocking Tim‐3 and p38, respectively

**Fig. 3:**
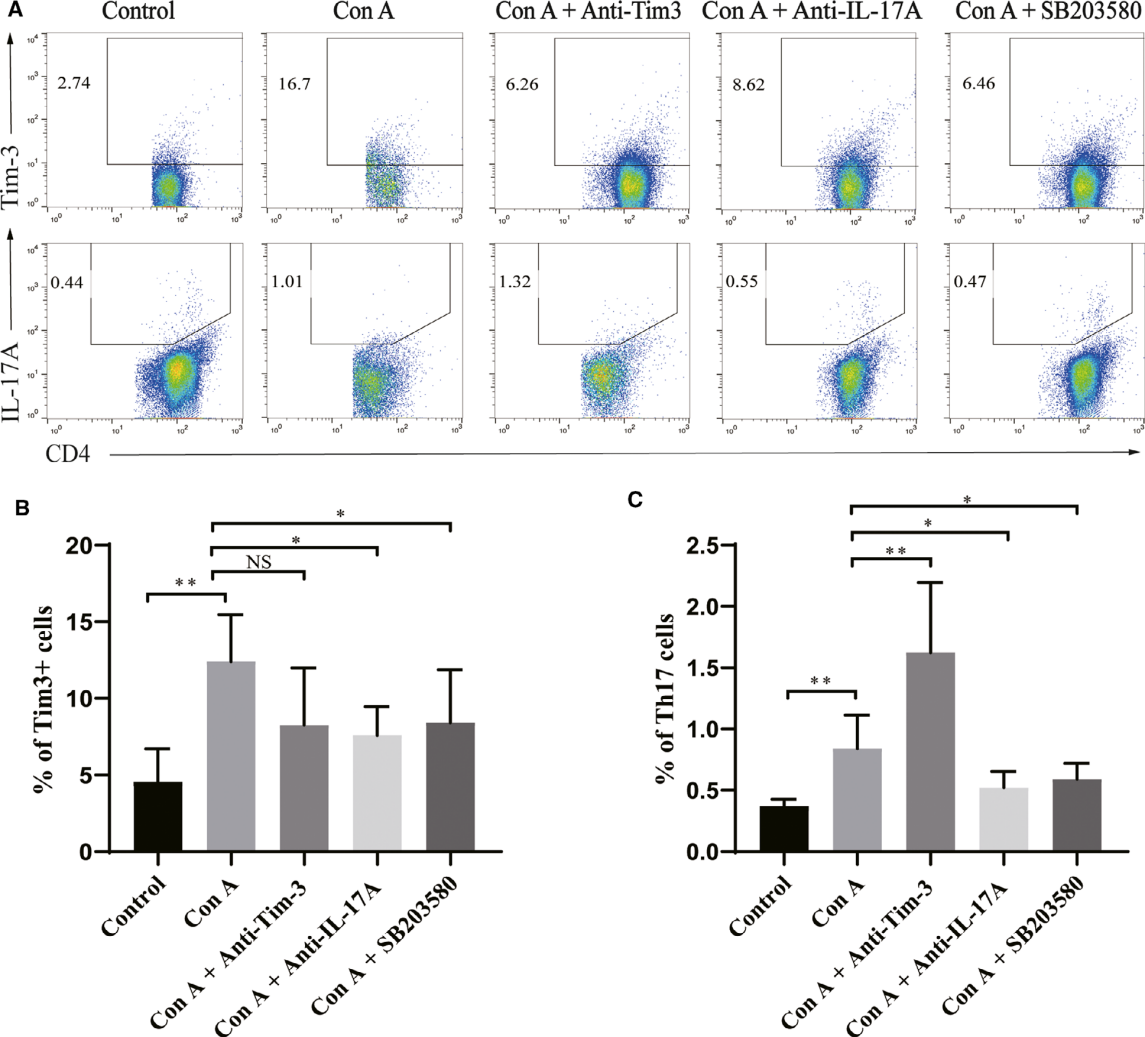
Tim‐3 suppressed AIH, and blockage of the p‐38 pathway decreased the frequency of Th17 cells. (A) Lymphocyte suspensions from mouse spleens were harvested after blocking and AIH induction. (B) Frequencies of CD4^+^ Tim‐3^+^ T cells were determined. (C) Frequencies of CD4^+^ IL‐17^+^ T cells were determined. One‐way analysis of variance was used to analyze the difference in results between multiple groups, and the LSD test was used for pairwise comparison. All values are reported as mean ± SD. *P < 0.05, and **P < 0.01, and NS indicates no significant difference. The experiments were performed in triplicate.

Flow cytometry analysis was performed to detect the proportion of Th17 cells and CD4^+^ Tim‐3^+^ T cells in mouse spleens to further confirm the blocking effect of antibodies against Tim‐3 and p38 and study the role of p38 in the regulatory effect of Tim‐3 on AIH. The results showed significantly higher frequencies of CD4^+^ Tim‐3^+^ T cells (12.4 ± 2.9%) and CD4^+^ IL‐17^+^ T cells (0.84 ± 0.25%) in the Con A‐alone group compared with those of the control group (CD4^+^ Tim‐3^+^ T cells; 4.6 ± 1.9%, *P* < 0.01; and CD4^+^ IL‐17^+^ T cells; 0.37 ± 0.05%, *P* < 0.01). The Tim‐3 blocking group showed a significant decrease in the proportion of CD4^+^ Tim‐3^+^ T cells (8.3 ± 3.2%) and an increase in CD4^+^ IL‐17^+^ T cells (1.6 ± 0.5%) compared with the mice receiving only Con A injection, indicating the significant molecular blocking effect. Furthermore, we noticed that the proportions of both CD4^+^ Tim‐3^+^ T cells (8.4 ± 3.2%) and CD4^+^ IL‐17^+^ T cells (0.59 ± 0.12%) in the p38‐blocked group and CD4^+^ Tim‐3^+^ T cells (7.6 ± 1.5%) and CD4^+^ IL‐17^+^ T cells (0.52 ± 0.13%) in the anti‐IL‐17A group were similarly reduced, suggesting that p38 affected the expression levels of Th17 cells and Tim‐3 (Fig. 3).

### Higher and lower levels of IL‐17A by blocking Tim‐3 and p38 signaling, respectively

**Fig. 4:**
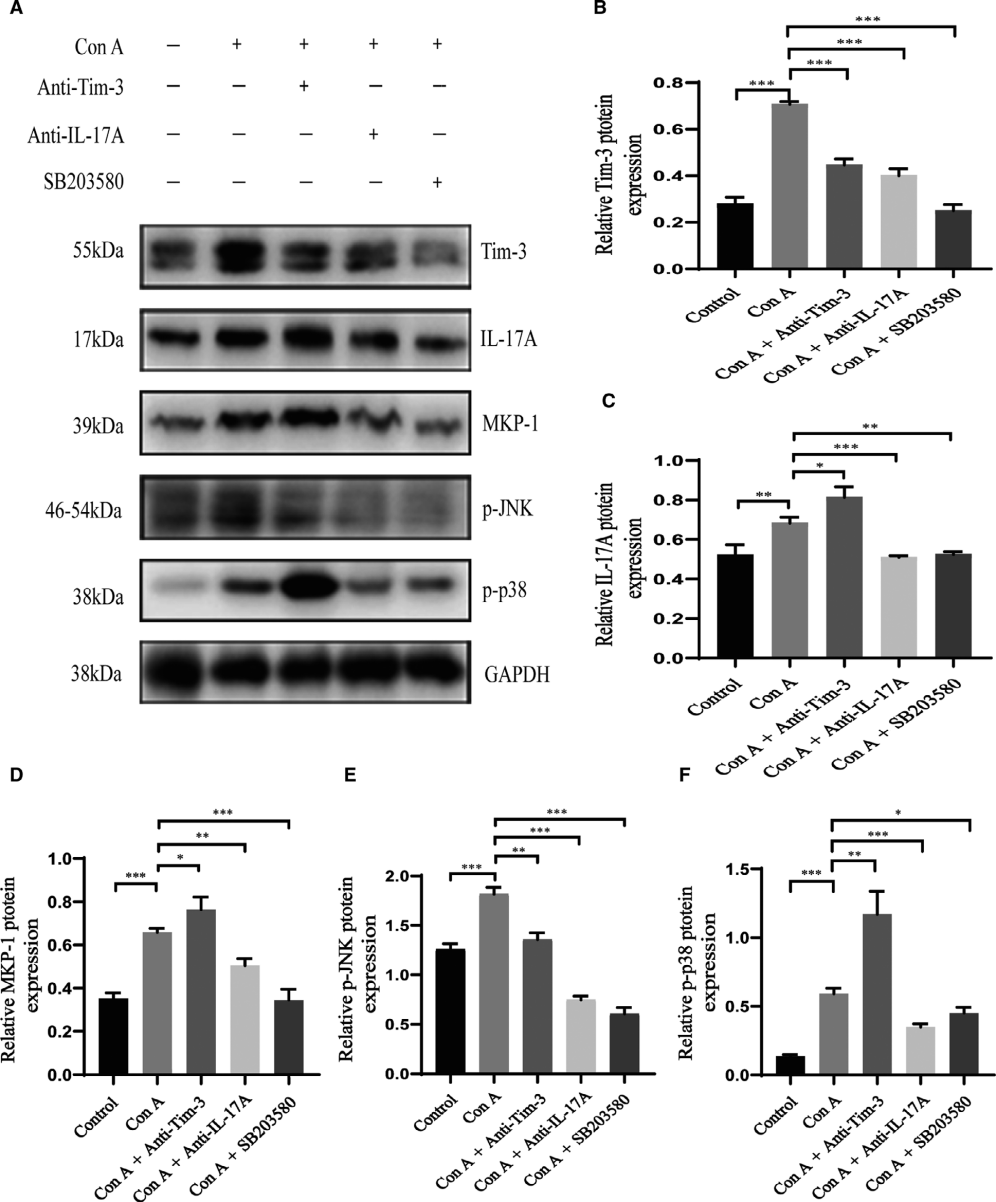
Tim‐3 regulated Th17 cells to suppress AIH through the p38/MKP‐1 pathway. (A) Western blot‐detected expression of Tim‐3, MKP‐1, p‐JNK, p‐p38, and GAPDH proteins in liver tissues. (B), (C), (D), (E), and (F) correspond to quantitative measurements of the band intensities determined by the Image J (version 1.52a; National Institutes of health, Bethesda, MD, USA). One‐way analysis of variance was used to analyze the difference in results between multiple groups, and the LSD test was used for pairwise comparison. All values are reported as mean ± SD, *P < 0.05, **P < 0.01, and ***P < 0.001. The experiments were performed in triplicate.

To further investigate the underlying mechanisms of the regulatory effects of Tim‐3 on AIH, the expression levels of IL‐17A, p‐JNK, and p‐p38 in mouse liver tissues in different groups were evaluated by western blot analysis. Consistent with the results obtained by testing the peripheral blood of patients with AIH, western blot analysis showed that the expression levels of IL‐17A, p‐JNK, and p‐p38 in liver tissues from the AIH mouse model (Con A alone) were significantly higher than in the control group (*P* < 0.01). Additionally, the differences in Tim‐3 and MKP‐1 protein levels in model animals were significantly higher than those in the control group (*P* < 0.001). Similarly, by blocking the Tim‐3 signaling pathway with anti‐Tim‐3 antibodies, the IL‐17A, MKP‐1, and p‐p38 expression levels were further upregulated upon Con A treatment, while those of p‐JNK were downregulated. These results suggest that the Tim‐3 pathway may have a regulatory effect on IL‐17A and p38. Furthermore, in the anti‐IL‐17A and p38 blocked groups, the levels of Tim‐3, IL‐17A, MKP‐1, p‐JNK, and p‐p38 were significantly downregulated (Fig. 4).

## Discussion

Autoimmune liver disease is a heterogeneous liver disease, defined and classified mainly on the basis of the cause, stage, and grade. The loss of tolerance to self‐antigens in the liver is considered a potential pathogenic mechanism in genetically susceptible individuals and may be triggered by environmental factors, such as pathogens and foreign substances [21]. Triggering factors, such as viral exposure, lead to inappropriate T‐cell‐mediated autoimmune responses against liver autoantigens. AIH, a condition found in genetically predisposed individuals, also leads to the loss of tolerance to self‐antigens and thus induces an inappropriate regulatory immune control [22].

There are several inducers used to establish AIH in mice, including Con A, high dosage of lipopolysaccharides (LPS), and D‐galactosamine with low dosage of LPS (GalN/LPS). Con A, also known as T‐cell mitogen, is a plant lectin extracted from broad beans that can cross‐link T cells with hepatic sinusoidal endothelial cell surface glycoproteins and MHC‐II molecules of Kupffer cells, promote the recruitment to and activation of CD4^+^ T cells and NK cells in the liver, and induce the excessive synthesis of cytokines, such as TNF‐α, IFN‐γ, IL‐1β, IL‐2, and IL‐6 [23]. According to Wang *et al*., the Con A‐induced AIH model is superior to the other two in several aspects, such as easier establishment since it requires only one inducer. Besides, Con A could induce significant changes in the transaminase levels while the LPS model failed, and also induce remarkable changes in serum levels of many inflammation‐relevant cytokines, which favors the research on the underlying mechanisms of AIH [24]. Therefore, in this study, we used Con A to establish an AIH mouse model from C57BL/6 mice. After injection of Con A, ALT and AST levels in mice were elevated, and liver sections showed the formation of vacuoles, loss of liver structure, and lymphocyte infiltration with varying degrees of inflammatory (grade 1–2) and pathological changes, indicating that the AIH model was successfully established. After blocking Tim‐3, ALT and AST concentrations were increased significantly, and obvious lymphocyte infiltration was noted, indicating that intact Tim‐3 must have a suppressive or restorative effect on AIH. According to Sakuishi *et al*., when the interaction between Tim‐3 and galectin‐9 is blocked *in vivo*, it leads to increased autoimmunity and loss of tolerance in experimental models. So Tim‐3 is considered a negative regulator [25]. In another study, after isolating CD4^+^ T cells from patients with MS and colitis, both autoimmune diseases, Tim‐3 expression and signal transduction defects were observed [26]. These results were consistent with our findings. In our experiment, the number of CD4^+^ Tim‐3^+^ T cells in human peripheral blood was higher in the normal group than in the AIH group. However, in the mouse model the expression of Tim‐3 protein in the Con A‐induced AIH group was higher than in the control group, as determined by western blot. As AIH is a chronic liver disease with immune dysfunction and administration of Con A to mice only induces acute liver injury, the changes in Tim‐3 levels in patients with AIH better reflect the real role of this protein in AIH. Therefore, the fact that Tim‐3 levels in the Con A group were higher than in the normal group, as determined by western blot, suggests that the rebound of Tim‐3 is higher.

Recent studies have found that the duration and severity of hepatitis may depend on the balance of regulatory T cells (Treg), Th17, Th1, and cytotoxic T cells (CTL) [27]. Of note, in AIH patients, the number of Treg cells was negatively correlated with disease activity indicators [28]. Treg cells express external nucleoside triphosphate diphosphate hydrolase 1 (also known as CD39). The reduction in Treg cells leads to the decreased hydrolysis of pro‐inflammatory nucleotides, and the effect on the inhibition of IL‐17 production by CD4+ T cells is weak [29]. On the other hand, Th17 cells secrete IL‐17, which can aggravate liver damage and promote the pathogenesis of autoimmune diseases in mice and humans. The number of γδT cells in patients with AIH is also increased. In particular, in the active phase, the expression of granzyme B effector cell molecules is directly related to the levels of biochemical indicators of liver injury, such as ALT and bilirubin. The molecules on liver cells enable them to act as antigen‐presenting cells, which contribute to the persistence of liver damage [30]. The number of liver Th17 cells in AIH patients increases with the severity of the disease. [31]. In our experiments, the expression of IL‐17A was significantly increased in both AIH patients and AIH model mice.

A variety of pathways play an irreplaceable role in AIH, including the MAPK pathway. After activation, JNK enters the nucleus (from the cytoplasm) and activates the c‐Jun transcription factor through the transphosphorylation of Ser73 and Ser63, which then interacts with the binding site of the transcription factor activator protein‐1 (AP‐1) located in the gene promoter region, and expressions of pro‐inflammatory genes and the consequent protein synthesis increase; for instance, the expression of TNF‐α, IL‐6, and IL‐8 is mediated through this pathway [32]. MAPK kinases 3 and 6 activate p38 MAPK, which can phosphorylate a range of transcription factors and promote the production of inflammatory factors. In addition to the regulation of MAPK phosphorylation by upstream kinases, the inactivation of MAPK in mammalian cells is also caused by the dual‐specific MKP (MAPK phosphatase family), which acts on two key phosphorylation sites in the activation loop of MAPK. Our experiments determined that the expression of p‐p38 gradually increased as the inflammation intensified, but the blocking of the p38 pathway significantly decreased the expression of the inflammatory factor IL‐17A. It was observed that MKP‐1 expression gradually increased in the normal, Con A, and anti‐Tim‐3 groups. MKP‐1 is responsible for the dephosphorylation of MAPK [33], thereby inhibiting inflammation. We found that the expression of MKP‐1 levels in human serum samples from the normal group was higher than in AIH patients. Therefore, the increased p‐p38 expression, as observed by western blot, led to an increase in MKP‐1 stress. To the best of our knowledge, very few studies measured serum levels of MKP‐1 and p‐p38 when investigating their roles in the development of a specific disease, since they are intracellular signaling proteins. But serum MKP‐1 and p38 levels were really detected by ELISA in some studies [34, 35], Our study showed that the difference of serum MKP‐1 levels between AIH and healthy people was also significant. By blocking Tim‐3, p38 was activated and MKP‐1 was inhibited, thus upregulating IL‐17A expression and promoting the development of AIH. Furthermore, although the p‐JNK levels in the Con A group were higher than in the control group, these decreased in the anti‐Tim‐3 group before the mouse model was established. Because the p‐JNK pathway is complex and interacts with other pathways, further research is needed to sort out the ramifications of Tim‐3 blocking.

In summary, Con A‐induced AIH activated p38 and inhibited MKP‐1 levels, thus activating CD4^+^ T cells to differentiate into Th17 cells, upregulating IL‐17A expression. Upregulated Tim‐3 expression exerted immunomodulatory effects by inhibiting the p38 pathway, reducing liver damage. Blocking the Tim‐3 pathway, activation of p38, and inhibition of MKP‐1 resulted in significantly increased IL‐17A levels and aggravated liver damage. The facts above indicate that Tim‐3 expression may regulate Th17 cells through the P38/MKP‐1 pathway to suppress autoimmune liver disease. The results obtained in this study provide new insights into the treatment of autoimmune liver disease.

## Conflict of interest

The authors declare no conflict of interest.

## Author contributions

ZY conceived and designed the study. HW, JX, MZ, and ST performed the experiments. HW was responsible for analyzing the data and writing the manuscript. ZY and JZ supervised the experiments and manuscript.

## Data Availability

The analyzed data sets generated during the present study are available from the corresponding author on reasonable request.
